# 628. Utilizing Cefepime Therapeutic Drug Monitoring for Patients at Risk of or Exhibiting Signs of Neurotoxicity

**DOI:** 10.1093/ofid/ofac492.680

**Published:** 2022-12-15

**Authors:** Joanna Au, Sumeet Jain, Amanda Lin, Evelyn Luo, Pranisha Gautam-Goyal, Patricia Saunders-Hao

**Affiliations:** North Shore University Hospital, Manhasset, New York; North Shore University Hospital, Manhasset, New York; North Shore University Hospital, Manhasset, New York; North Shore University Hospital, Manhasset, New York; Zucker School of Medicine at Hofstra/Northwell, Manhasset, New York; North Shore University Hospital, Manhasset, New York

## Abstract

**Background:**

Increasing risk of neurotoxicity has been observed with increasing cefepime levels, potentially due to the competitive inhibition of GABA receptors in the central nervous system (CNS). The suggested threshold is ≥20 mg/dL. Risk factors include decreased clearance with renal impairment, or increased CNS penetration with blood-brain barrier dysfunction. This study analyzes the association between high trough levels and risk factors for cefepime-induced neurotoxicity (CIN), and the utility of therapeutic drug monitoring (TDM) in guiding cefepime therapy.

**Methods:**

This was a prospective single-arm observational study. Eligible patients had signs of potential CIN or had risk factors including cefepime ≥4 g/day for ≥4 days, renal impairment, CNS disease, and/or co-administration of neurotoxic medications. Interventions for discontinuation or dose adjustment were made for levels ≥20. The primary outcome was the incidence of troughs ≥20 in patients with possible signs of or risk factors for CIN. The secondary outcome was the proportion of patients whose therapy is altered in response to TDM.

**Results:**

A total of 20 patients were included. Reasons for inclusion were 15 with renal impairment, 10 had signs of neurotoxicity, and 5 were included to confirm therapeutic dosing. Patients could have multiple risk factors for CIN. Ten of 20 had a trough ≥20 (median 19). Six of 10 with signs of neurotoxicity had a trough ≥20 (OR 2.25, 95% CI 0.38-13.47; p=0.66) and 10 of 15 with renal impairment had a trough ≥20 (RD 0.67, 95% CI 0.13-0.89; p=0.0325). Cefepime was renally dose adjusted in 7 of 10 with a trough ≥20. Of the total 20 patients, 16 required interventions of which 12 were confirmed by the trough result.

**Conclusion:**

Renal impairment was found to be a statistically significant risk factor for CIN, even when cefepime was renally dose adjusted. These patients may benefit from TDM. TDM allowed for confirmation of interventions and further interventions were made based on trough levels. Despite a small sample size, cefepime TDM has the potential to identify patients at risk for CIN and may assist in making dose adjustments to improve patient safety.

**Disclosures:**

**All Authors**: No reported disclosures.

